# *In vitro* expansion impaired the stemness of early passage mesenchymal stem cells for treatment of cartilage defects

**DOI:** 10.1038/cddis.2017.215

**Published:** 2017-06-01

**Authors:** Tongmeng Jiang, Guojie Xu, Qiuyan Wang, Lihui Yang, Li Zheng, Jinmin Zhao, Xingdong Zhang

**Affiliations:** 1 Guangxi Engineering Center in Biomedical Materials for Tissue and Organ Regeneration, The First Affiliated Hospital of Guangxi Medical University, Nanning 530021, China; 2 Department of Orthopaedics Trauma and Hand Surgery, The First Affiliated Hospital of Guangxi Medical University, Nanning 530021, China; 3 Center for Genomic and Personalized Medicine, Guangxi Medical University, Nanning 530021, China; 4 School of Nursing, Guangxi Medical University, Nanning 530021, China; 5 Collaborative Innovation Center of Guangxi Biological Medicine, The First Affiliated Hospital of Guangxi Medical University, Nanning 530021, China; 6 Guangxi Key Laboratory of Regenerative Medicine, The First Affiliated Hospital of Guangxi Medical University, Nanning 530021, China; 7 National Engineering Research Center for Biomaterials, Sichuan University, Chengdu 610064, China

## Abstract

*In vitro* cultured autologous mesenchymal stem cells (MSCs) within passage 5 have been approved for clinical application in stem cell-based treatment of cartilage defects. However, their chondrogenic potential has not yet been questioned or verified. In this study, the chondrogenic potential of bone marrow MSCs at passage 3 (P3 BMSCs) was investigated both in cartilage repair and *in vitro,* with freshly isolated bone marrow mononuclear cells (BMMNCs) as controls. The results showed that P3 BMSCs were inferior to BMMNCs not only in their chondrogenic differentiation ability but also as candidates for long-term repair of cartilage defects. Compared with BMMNCs, P3 BMSCs presented a decay in telomerase activity and a change in chromosomal morphology with potential anomalous karyotypes, indicating senescence. In addition, interindividual variability in P3 BMSCs is much higher than in BMMNCs, demonstrating genomic instability. Interestingly, remarkable downregulation in cell cycle, DNA replication and mismatch repair (MMR) pathways as well as in multiple genes associated with telomerase activity and chromosomal stability were found in P3 BMSCs. This result indicates that telomerase and chromosome anomalies might originate from expansion, leading to impaired stemness and pluripotency of stem cells. *In vitro* culture and expansion are not recommended for cell-based therapy, and fresh BMMNCs are the first choice.

Identifying appropriate cell sources is a challenge in cell-based therapies for cartilage repair. As an established strategy for cartilage restoration, autologous chondrocyte implantation (ACI) has received intense attention and has yielded encouraging results. However, donor site morbidity and chondrocyte dedifferentiation during expansion *in vitro* have limited the application of ACI. Alternative repair strategies based on mesenchymal stem cells (MSCs) are highly recommended for clinical applications due to their high proliferation, high plasticity and multipotency. Importantly, bone marrow MSCs (BMSCs) may instead chondrocytes based on non-inferiority in clinical outcomes.^1, 2^ Tissue-engineering technologies are also integrated in stem cell-based therapies. Biodegradable scaffolds such as collagen have been widely used in conjunction with MSCs to aid cell delivery and support chondrogenic differentiation, functional extracellular matrix formation and three-dimensional tissue development.^3, 4^

For MSC-based strategies, expansion *in vitro* is always required to generate sufficient stem cells for transplantation.^5^ It is generally accepted that this type of MSC preparation is acceptable and is not only approved by the European regulation (European Commission 1394/2007) but also by the Food & Drug Administration in the USA.^6^ Clinical recommendations for use of MSCs is usually at 3–5 passages.^7, 8^ However, there are complications: (1) a two-step surgical procedure is painful and time-consuming (often 3–6 weeks);^9, 10^ (2) unexpected risks can occur during expansion, including contamination, lack of phenotype, and reduction in efficiency, potentially leading to therapy failure; and (3) robust production processes must be developed by optimizing culture variables, cell seeding density, physiochemical environment, and subculture protocols. So far, the disadvantages of the MSC strategies requiring expansion *in vitro* are only technological limitations.

The pluripotency of early passage MSCs has not yet been contradicted, although it is well known that cell differentiation and function decline with passaging. First-passage MSCs have a markedly diminished proliferation rate and gradually lose their multipotency, thus greatly reducing bone-forming efficiency *in vivo* compared with the fresh bone marrow cells.^11^ MSCs at passages 1–2 are superior to those at passages 3–4 and markedly improve survival in patients who receive stem cell-based therapy.^12^ Passage 2 or 3 cells have much weaker pluripotency than passage 1 cells.^12^ These results indicate that expansion might attenuate the stemness of MSCs, thereby contributing to reduced therapeutic potential. It has been verified that monolayer culture greatly influences cell behavior,^13^ resulting in cell senescence and impairing multipotency.^14^ In serial passage of MSCs, telomere activity and chromosome heteromorphosis increase over time.^15, 16, 17^ In addition, *in vitro* conditions, including culture media^18^ and hypoxia, may be obstacles for MSC bioactivity and clinical application.^19^ The direction of MSC differentiation cannot be precisely controlled, and completely pure MSCs cannot be obtained.^20^ Several studies have reported that freshly isolated bone marrow mononuclear cells (BMMNCs) might be an alternative to culture-expanded MSCs for bone tissue engineering^21, 22^ and repair of full-thickness osteochondral defects.^23^ However, a comparison of early passage MSCs and BMMNCs has not been reported.

We hypothesized that expansion based on flat-surface cell culture systems have adverse effects on the differentiation capabilities of MSCs and, consequently, MSC-based therapy. MSCs at early passage are questionable regarding their stemness. The objective of this study was to investigate the chondrogenic potential and therapeutic effect of early passage BMSCs at passage 3 (P3 BMSCs) compared to BMMNCs. Collagen hydrogels were used as the vehicle both *in vivo* and *in vitro*. A follow-up study of cartilage defects was performed after 4 to 52 weeks of therapy, and further expansion-driven genetic alterations were studied.

## Results

### P3 BMSC- and BMMNC-based therapy to treat cartilage defects

**Figure 1:**
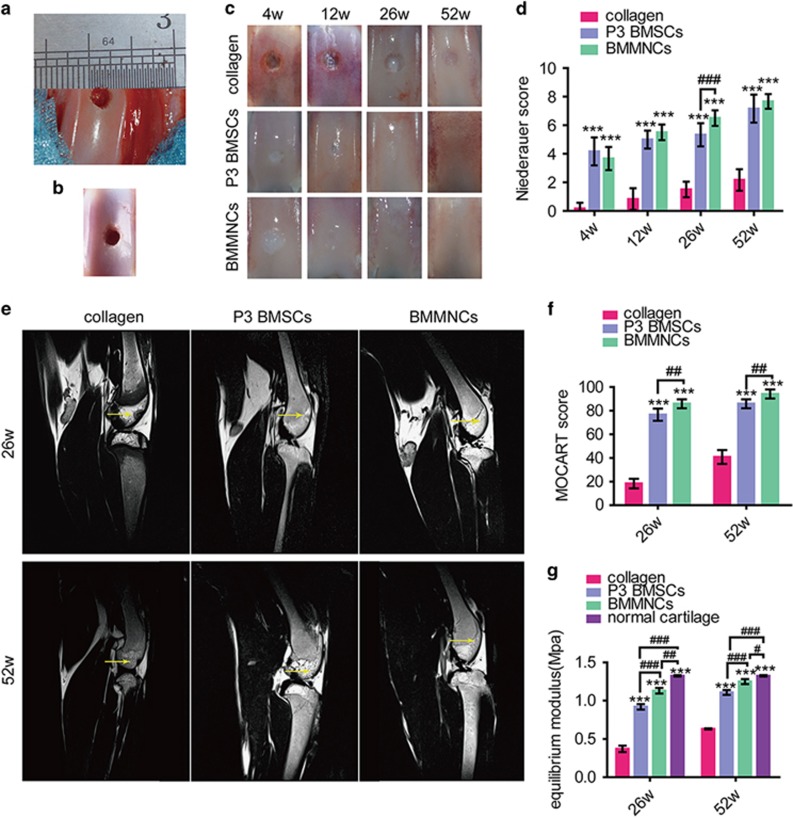
Macroscopic findings, MRI, and mechanical evaluation of repaired cartilage in *vivo*. (**a**) A 4-mm chondral defect was created in the medial area of the patella groove. (**b**) Macroscopic finding of the untreated defect at 52 week post-operation. (**c**) Defects filled with collagen I, BMMNCs/collagen I and P3 BMSCs/collagen I at different post-transplantation time, respectively. (**d**) Niederauer scoring system were used at each time point; *n*=6. (**e**) The surface and structure of the repaired tissues were examined by MRI based on the signal intensity of the repaired tissue and the subchondral bone status. (**f**) MOCART score response to the MRI results; *n*=6. (**g**) Mechanical evaluation was performed at 26 weeks and 52 weeks after transplantation; *n*=6. Error bars, mean±S.D. *, ^#^*P*<0.05; **^, ##^*P*<0.01; ***^, ###^*P*<0.001

To validate our hypothesis *in vivo*, we established a model (a 4-mm diameter defect in the patellar groove of rabbit femurs) by drilling (Figure 1a). The defects did not self-heal up to 52 weeks after surgery (Figure 1b). No malformation was observed in the engineered cartilage at all time points (Figure 1c). The bottom of the defects could be observed over time, whereas defects in two cell-treated groups formed cartilage-like tissue gradually. The margins were detectable, and the defects were covered with white opaque tissue until week 26 for P3 BMSC-based therapy but week 12 for BMMNC-collagen implantation. The defect was barely discernible after 26 weeks of therapy with BMMNCs, whereas traceable marks were observed at 26 weeks for the P3 BMSCs. Furthermore, a Niederauer score was used to evaluate the gross morphology of the engineered cartilage (Figure 1d). The P3 BMSC group had lower scores than BMMNC group upon gross morphological evaluation at 12, 26 and 52 weeks but not at 4 weeks. The lowest scores were observed for collagen therapy alone.

The surface and structure of the repaired tissues were examined via magnetic resonance imaging (MRI) based on the signal intensity and the subchondral bone status (Figure 1e). In the images, defects appeared dark against bright normal cartilage in the collagen group at both 26 and 52 weeks, indicating the formation of non-hyaline cartilage. Engineered tissue based on P3 BMSCs exhibited intermediate signals adjacent to high-signal cartilage, whereas tissue formed from BMMNCs exhibited higher signals near the surrounding cartilage. At 52 weeks, the signal in the BMMNC-engineered cartilage was nearly identical to that in the surrounding cartilage. Accordingly, the MOCART scores were significantly lower in P3 BMSC group than in BMMNC group (*P*<0.01) (Figure 1f).

To measure the inherent compressive stiffness of the solid matrix, we assessed compressive modulus of the engineered tissues (Figure 1g) with normal rabbit cartilage as the control. The compressive modulus of the repaired tissues increased from 26 to 52 weeks in all groups. The BMMNC-engineered cartilage was significantly stiffer (*P*<0.001) than the other two groups and was similar to normal cartilage at 52 weeks. Cartilage repaired using only collagen exhibited the lowest mechanical strength, indicating the formation of fibrocartilage.

**Figure 2:**
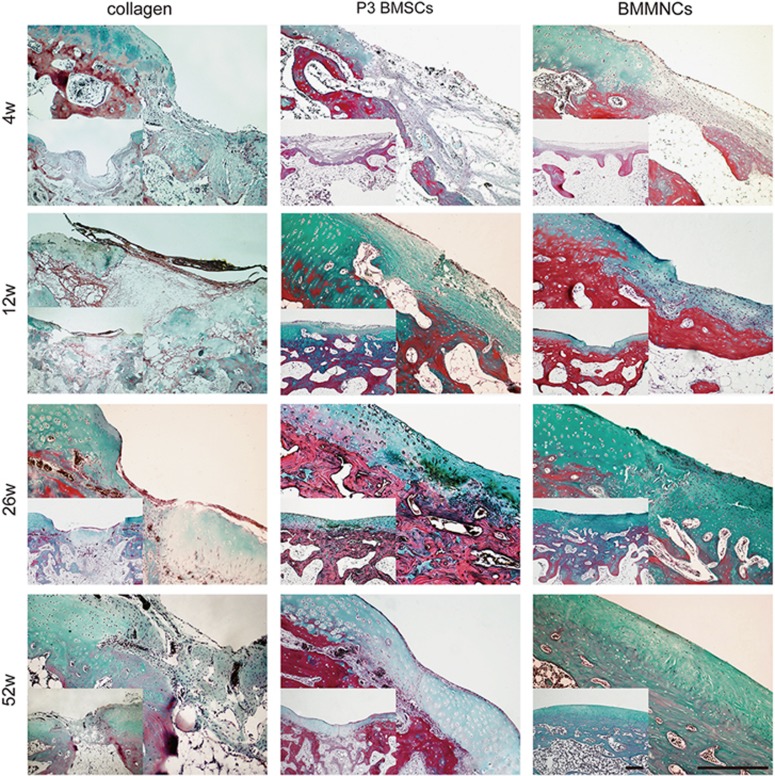
Masson trichrome staining of repaired cartilage *in vivo* at different post-transplantation time, respectively. Scale bar: 400 *μ*m

**Figure 3:**
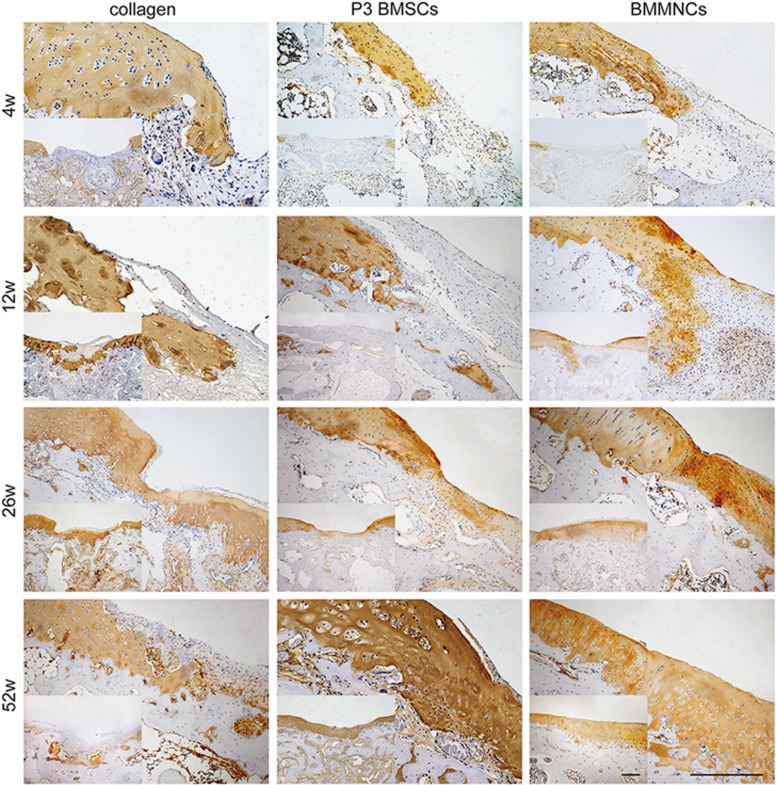
Immunohistochemical analysis for collagen type I of repaired cartilage *in vivo* at different post-transplantation time, respectively. Scale bar: 400 *μ*m

Histological analysis was performed with Masson trichrome staining (Figure 2), hematoxylin–eosin (HE) staining (Supplementary Figure S1), Safranin-O staining (Supplementary Figure S2) and immunohistological detection (Figure 3). In collagen group, the neocartilage was primarily composed of fibrous tissue with a modicum of distributed cells, and the interface was loose and detached during the process. A distribution of some inflammatory cells was observed only at 4 weeks. However, staining for collagen type II, an indicator of cartilage differentiation, was nearly absent in the repaired tissue. The newly formed tissue gradually progressed from fibrous tissue to hyaline cartilage-like tissue following cell-based therapy. BMMNC-engineered cartilage exhibited more uniform and compact tissue with more round cells in the lacuna compared to P3 BMSC-engineered cartilage 12 weeks after surgery. At 52 weeks, the boundary between the neocartilage and the original tissue was barely discernible in the BMMNC group but remained obvious in P3 BMSC group. The morphology of BMMNC but not P3 BMSC-engineered tissue resembled normal cartilage. Moreover, lower glycosaminoglycan (GAG) production and more negatively stained area of collagen II were observed in P3 BMSC group than in BMMNC group after 12 weeks. An exception occurred at 4 weeks, when P3 BMSC group exhibited a superior performance to BMMNC group.

**Figure 4:**
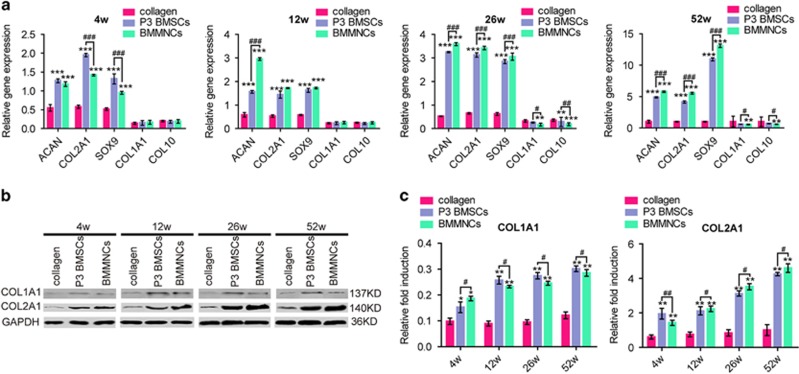
Cartilage associated gene/protein expression of repaired cartilage *in vivo.* (**a**) Real-time PCR for *COL1A1*, *COL2A1*, *COL10*, *SOX9* and *ACAN* of the defects at 4 weeks, 12 weeks, 26 weeks and 52 weeks after transplantation; *n*=6. (**b**) Western blot results of COL1A1 and COL2A1 at different post-transplantation time, respectively. (**c**) Normalized expression of COL1A1 and COL2A1 in response to WB analysis; *n*=6. Error bars, mean±S.D. *^, #^*P*<0.05; **^, ##^*P*<0.01; ***^, ###^*P*<0.001

The expression of specific markers in the engineered cartilage was measured with real-time PCR (Figure 4a) and WB analysis (Figures 4b and c), respectively. The expression of cartilage-specific genes, such as *ACAN*, *COL2A1* and *SOX9*, was much higher in two cell-treated groups than in collagen group. The expression of *ACAN*, *COL2A1* and *SOX9* was significantly lower in P3 BMSC group than in BMMNC group at week 12, 26 and 52. The opposite pattern was observed at 4 weeks. The levels of *COL1A1* (a fibrocartilage marker) and *COL10* (an indicator of hypertrophy) were higher in P3 BMSC groups than in BMMNC groups at week 26 and 52. The protein expression levels (Figure 4c) confirmed the findings of PCR analysis (Figure 4a).

### Chondrogenic potential of P3 BMSCs and BMMNCs in 3D collagen

**Figure 5:**
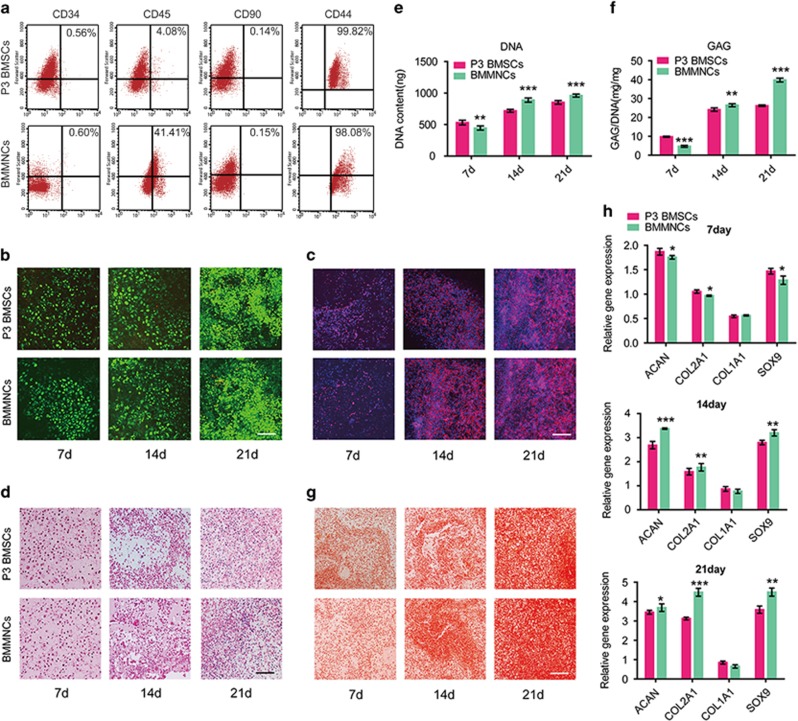
Expansion of MSCs contributes to different results in cartilage engineering. (**a**) Flow cytometric analysis was performed to characterize the phenotypes of the P3 BMSCs and uncultured BMMNCs. (**b**) Cell viability analysis was performed to detect live/dead cells. Viable cells appear green, whereas dead cells appear red. Scale bar: 500 *μ*m. (**c**) Cytoskeletal morphology was assessed using phalloidin staining of the actin cytoskeleton and cell nuclei via Hoechst 33258 by confocal microscopy. Scale bar: 500 *μ*m. (**d**) H&E staining. Scale bar: 500 *μ*m. (**e**) Cell proliferation was measured based on the DNA content after 7, 14 and 21 days of culture in 3D collagen hydrogels and chondrogenic supplements; *n*=5. (**f**) The GAG production was normalized to the total DNA production to evaluate the biosynthetic activity; *n*=5. (**g**) Safranin-O staining. Scale bar: 500 *μ*m. (**h**) Genes related with chondrogenesis (*COL1A1*, *COL2A1*, *COL10*, *SOX9* and *ACAN*) were detected by real-time PCR after 7, 14 and 21 days of culture in 3D collagen hydrogels and chondrogenic supplements; *n*=5. Error bars, mean±S.D. *P<0.05; **P<0.01; ***P<0.001

For *in vitro* study, flow cytometric analysis was performed to characterize the phenotypes of P3 BMSCs and BMMNCs (Figure 5a). P3 BMSCs and BMMNCs had similar surface phenotypes with equivalent expression of CD34, CD44, and CD90. However, BMMNCs expressed CD45 at higher levels than P3 BMSCs.

Cell viability analysis was performed to detect live/dead cells. Viable cells appear green, whereas dead cells appear red (Figure 5b). The proportion of live cells increased over time in both groups. A remarkable increase in cell number was apparent from day 14 to day 21, with numerous cell clusters forming by day 21. A small number of dead cells was present followed by a large number of viable cells, possibly due to oxygen and nutrient diffusion limitations through collagen I matrix or an increase in the consumption of available nutrients. Comparatively, fewer living cells were present in P3 BMSC group than in BMMNC group at 14 and 21 days.

Cytoskeletal morphology was assessed using phalloidin staining of the actin cytoskeleton and Hoechst 33258 staining of cell nuclei followed by confocal microscopy (Figure 5c). The number of intranuclear actin filaments increased over time in both P3 BMSCs and BMMNCs. In comparison, P3 BMSCs in collagen contained less actin and appeared to be larger than BMMNCs in collagen on days 14 and 21. On day 7, the difference between the two groups was not marked.

We performed HE staining to investigate the cell distribution and evaluate the chondrocyte characteristics within scaffolds (Figure 5d). Round-shaped chondrocyte-like cells and lacuna structures were distributed homogenously in both P3 BMSC and BMMNC groups. In P3 BMSC group, fewer round cells with chondrocyte characteristics were present and the formation of lacuna structures was less obvious compared with BMMNC group on day 14 and 21.

Cell proliferation was measured based on the DNA content (Figure 5e). Both P3 BMSCs and BMMNCs exhibited a time-dependent increase in the number of cells. The number of P3 BMSCs was greater than that of BMMNCs on day 7, but opposite results were observed on day 14 and 21. GAG production also gradually increased over time in both groups (Figures 5e and f). Quantitatively, GAG production in BMMNCs was significantly improved compared to P3 BMSCs at day 14 and 21 but not at initial culture period of 7 days (Figure 5f). GAG secretion was 51.82% lower in P3 BMSCs than in BMMNCs at day 21. Safranin-O staining (Figure 5g) was performed to measure the synthesis and secretion of GAG. Consistent with the GAG content, Safranin-O staining indicated a less homogeneous distribution of cells embedded in less abundant GAGs in P3 BMSCs than in BMMNCs on day 14 and 21. By contrast, more cells and more intense staining were observed in P3 BMSCs than in BMMNCs on day 7.

Chondrogenic marker genes were detected with real-time PCR (Figure 5h). After 7 days of culture, the expression levels of *ACAN*, *COL2A1* and *SOX9* were higher in P3 BMSCs than in BMMNCs. However, at later stage, BMMNCs in the collagen hydrogels expressed higher levels of the early chondrogenic transcription factor *SOX9* (*P*<0.01) and the late genes *ACAN* (*P*<0.05) and *COL2A1* (*P*<0.01) compared with the P3 BMSCs in collagen. There was no significant difference in the fibrocartilage marker *COL1A1* between groups. *COL10* (an indicator of hypertrophy) was not detected in any of the groups.

### Telomerase activity and karyotype analysis of P3 BMSCs and BMMNCs

**Figure 6:**
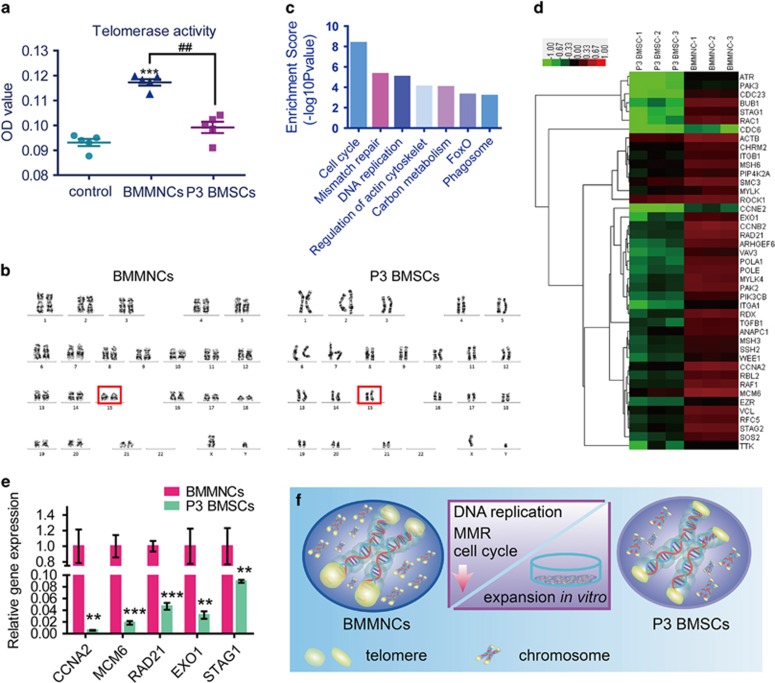
Expansion of MSCs contributes to different results in cartilage engineering and its mechanism. (**a**) Telomerase activity of P3 BMSCs and BMMNCs; *n*=5. Error bars, mean±S.D. *^, #^*P*<0.05; **^, ##^*P*<0.01; ***^, ###^*P*<0.001. (**b**) G-banded karyotype of BMMNCs, 44, XY. G-banded karyotype of P3 BMSCs, 44, XY, add (15p). Red boxes indicate the difference. (**c**) Pathway enrichment analysis of downregulated associated with MSCs expansion. (**d**) Hierarchical cluster analysis of BMMNCs and P3 BMSCs at 0d and 21d. (**e**) Real-time PCR confirmation of the vital genes (*CNNA2*, *MCM6*, *RAD21*, *EXO1* and *STAG1*) in the network; *n*=5. Error bars, mean±S.D. *P<0.05; **P<0.01; ***P<0.001. (**f**) 2D expansion potentially result in low telomerase activity (telomere curtail) and chromosome abnormality by downregulation of cell cycle, DNA replication and MMR signaling pathway

To examine the effect of culture expansion on the senescence of MSCs, we observed the level of telomerase activity. BMMNCs showed higher telomerase activity than the control, indicating preserved juvenescence. However, the relative telomerase activity of P3 BMSCs was decreased to 18.18% compared with BMMNCs, which revealed that 2D expansion may contribute to the senescence of MSCs (Figure 6a).

To investigate the effects of culture expansion on genomic integrity, we analyzed the karyotype by G-banding in BMMNCs and P3 BMSCs (Figure 6b). All of the BMMNCs were normal, whereas 88% of P3 BMSCs had a normal diploid karyotype (2n, *n*=22). Comparatively, chromosomal modality and structure of P3 BMSCs and BMMNCs were quite different. The chromosomes were morphologically stout in BMMNCs but slim in P3 BMSCs. One anomalous karyotype was found in P3 BMSCs on chromosome 15 [44, XY, add (15p)], with an additional chromosome observed at the short-arm site of the chromosome 15 shown on the right in Figure 6b.

### Comparative profiling of P3 BMSCs and BMMNCs

To determine whether expansion-induced genetic mutations, we compared P3 BMSCs and BMMNCs before culture and after 21 days of culture in 3D collagen using a microarray analysis. The temporal patterns of activated genes associated with MSC expansion are presented in Figure 6c. Cell cycle, DNA replication and mismatch repair (MMR) signaling pathways, which are vital for MSC expansion, were significantly differentially expressed between P3 BMSCs and BMMNCs.

Gene ontology enrichment analyses of BMMNCs and P3 BMSCs after 21 days of culture in 3D collagen and chondrogenic supplement were also performed. BMMNCs or P3 BMSCs exhibited no enrichment in the cell cycle, DNA replication and MMR pathways after being cultured in 3D collagen and chondrogenic supplement.

The differentially expressed genes in signaling pathways associated with expansion are listed in Table 1. A total of 33 genes affected by cell expansion *in vitro* were found to be involved in the cell cycle pathway, 10 genes in MMR, and 12 genes in the DNA replication pathway. The corresponding molecules in these pathways were investigated by hierarchical clustering analysis (Figure 6d). The results indicated that these molecules might contribute to changes in MSC pluripotency.

To confirm the reliability of the microarray data, we examined the expansion-induced expression of several critical genes using Real-time PCR (Figure 6e). *CCNA2*, *MCM6*, *RAD21*, *EXO1* and *STAG1* expression was significantly downregulated after MSC expansion *in vitro*.

### Human data

**Figure 7:**
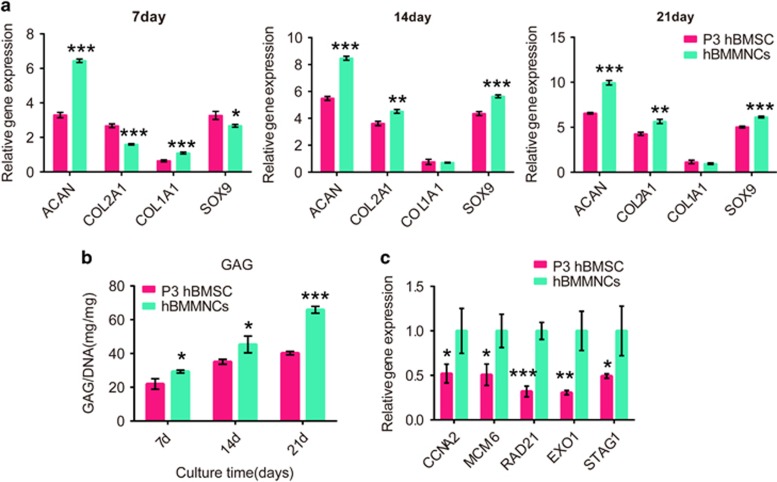
Expansion also affects chondrogenic differentiation of human MSCs *in vitro*. (**a**) Genes related to chondrogenesis (*COL1A1*, *COL2A1*, *COL10*, *SOX9* and *ACAN*) were detected using real-time PCR after 7, 14 and 21 days of culture in 3D collagen hydrogels and chondrogenic supplements; *n*=3. (**b**) The GAG production was normalized to the total DNA production to evaluate biosynthetic activity; *n*=3. (**c**) Real-time PCR confirmation of the expression of vital genes (*CNNA2*, *MCM6*, *RAD21*, *EXO1* and *STAG1*) in the network; *n*=3. Error bars, mean±S.D. *P<0.05; **P<0.01; ***P<0.001

To confirm the above findings, we compared the chondrogenic potential of P3 hBMSCs and hBMMNCs in 3D collagen and chondrogenic supplements. Interestingly, we found that the expression of chondrocyte-specific markers such as *ACAN*, *COL2A1* and *SOX9* was higher in hBMMNC group than in P3 hBMSC group at all time points (Figure 7a). In addition, the GAG quantity was also consistent with the mRNA expression of *ACAN* (Figure 7b). No telomerase activity was detected in either P3 hBMSCs or hBMMNCs. However, the *CCNA2*, *MCM6*, *RAD21*, *EXO1* and *STAG1* expression was significantly downregulated after MSC expansion *in vitro* (Figure 7c).

## Discussion

Our study verifies that compared with BMMNCs and P3 BMSCs exhibit inferior bioactivity and impaired therapeutic effectiveness in cartilage repair when combined with a 3D matrix. The stemness and differentiation ability of early passage BMSCs may be weakened by monolayer culture.

The impaired pluripotency of MSCs after expansion *in vitro* was verified through long-term observation of cartilage defect treatments. Massive lapin models are frequently used for research on cartilage tissue engineering.^24, 25, 26^ The most common width of experimental chondral defects in rabbit models is ~3 mm, the size at which intrinsic repair processes are predicted to reliably fail. This size also allows transplantation studies.^27^ In this study, a 4-mm diameter cartilage defect was created that could not be self-healed. In cell-based therapy, BMMNCs produced an inferior quality of repaired tissue in the early stage (at 4 weeks) compared to the P3 BMSCs. The proportion of cells with stem cell capabilities required for chondrogenesis may have been lower in BMMNC population than in P3 BMSC population after purification by expansion. However, as time progressed, this small number of progenitor cells exhibited superior differentiation ability compared to P3 BMSCs. Alternatively, the extracted BMMNCs may contain a heterogeneous population of cells (that is, MSCs, HSCs, EPCs, blood cells), and the variety of cytokines and growth factors secreted by these cells may have exerted synergistic effects on cartilage regeneration.^28^ The heterogeneous cell population in BMMNCs may have encouraging instead of inhibitory effects on hyaline cartilage formation. However, the presence of too many heterogeneous cells, such as blood cells in MSCs, may be harmful^29^ (for example, bone marrow without the purification procedure by microfracture). Following microfracture, the repaired tissue is largely fibrocartilage rather than hyaline cartilage^30^ due to high cell heterogeneity and insufficient recruitment of MSCs from the bone marrow.^31, 32^ Even concentrated bone marrow may form fibrocartilage in articular cartilage lesions in clinic.^33^ Thus, the extraction of BMMNCs is a priority for MSC therapy in clinic and can provide sufficient progenitor cells, although samples may be heterogeneous, and thus, BMMNCs may be mixed with a modicum of heterogeneous cells.

Although it is generally accepted that expansion *in vitro* is necessary to obtain sufficient MSCs. A total of 10^6^ mononuclear cells were isolated from 10 mL of bone marrow, sufficient for a cartilage defect 4-mm in diameter. The average density of mononuclear cells in human bone marrow is (123±74) × 10^8^ cells/ml.^34^ Chondrocytes account for only 1–2% of cartilage volume,^35^ and MSC therapy is suggested at a density of (5.8±2.3) × 10^6^ cells/ml.^36^ Thus, BMMNCs are mathematically sufficient for cartilage repair. Moreover, BMMNCs exhibited MSC markers that were similar to P3 BMSCs. MSCs are characterized by a lack of hematopoietic markers (CD34^−^ and CD45^−^) and a typical presence of CD44 surface antigen^37^ *in vitro*. CD90^−^ is specifically reported in rabbit MSCs.^38^ The BMMNCs presented typical MSC characteristics with the exception of HSC marker CD45, which suggests that some portion of BMMNCs might also have blood cell characteristics.

Prior 2D expansion also decreased chondrogenesis in 3D-culture system *in vitro*. The *in vitro* culture model was designed to mimic the cartilage environment by introducing a 3D collagen hydrogel and a chondrogenic supplement. For stem cell-based therapy, scaffolds are necessary^39, 40^ and have been suggested to be superior to cell therapy.^41^ Specifically, collagen gels mimic natural cartilage because they are highly hydrated 3D networks.^42^ During mid to late period (14–21days), BMMNCs exhibited greater chondrogenesis than P3 BMSCs, as evidenced by upregulated expression of chondrogenic markers. During initial stage of chondrogenesis (7 days), the chondrogenic ability of BMMNCs was inferior to that of P3 BMSCs. This suggests that heterogeneous population of BMMNCs was unfavorable for chondrogenesis only during initial stage. However, long-term effect of BMMNCs on chondrogenesis was advantageous compared to P3 BMSCs. In regard to human data, it showed that hBMMNCs are better than P3 hBMSCs for chondrogenesis at all time points. Even though a vast number of reports have demonstrated that human MSCs can be passaged more than three times and still retain multipotency,^43^ we still posit that uncultured hBMMNCs are the first choice for cartilage repair.

Although BMMNCs have a heterogeneous cell population, interindividual differences are not as notable as they are in P3 BMSCs, which are more suitable for clinical use. Comparative profiling of BMMNCs with P3 BMSCs provides insights into the changes that MSCs undergo during expansion. Important genes cross-linking were analyzed via clustering in the heat map. The differences among BMMNC group were minor. However, notable deviations were observed among P3 BMSCs, indicating that *in vitro* expansion greatly increased the genomic instability and heterogeneity of MSCs.

Interestingly, we observed an association between 2D expansion and mutations in genes associated with cell cycle, DNA replication and MMR, which are all related to telomerase activity and chromosomal stability.^44^ Telomeres are structures that protect DNA ends from degradation, DNA replication-associated attrition and stem cell self-renewal,^45^ and they participate in cell cycle, DNA replication and MMR processes that have key roles in maintaining chromosomal stabilization, accounting for stem cell pluripotency and self-renewal.^46^ Compared with BMMNCs, P3 BMSCs presented a decay in telomerase activity and a change in anomalous chromosome, indicating senescence. This was in accordance with change in cell cycle, DNA replication and MMR pathways, indicating that 2D expansion potentially leads to transcriptional downregulation of telomerase induced by preternatural DNA replication (Figure 6f). In the stem cell cycle, regulators like *CCNA2* and minichromosome maintenance (MCM) influence features of chromosomes in stem cells.^47^ As a component of MCM complex,^48^ *MCM6* is assembled on chromatin during DNA replication to enhance stem cell self-renewal and differentiation ability.^49, 50^ Downregulation of *CCNA2* and MCM6 indicated a chromosomal dismantlement at early stage in MMR signal transduction cascade,^50^ leading to induced multi-potent inefficiency. Other factors that affect chromosome segregation, MMR and chromosome morphology include cohesin complex (including *STAG1*).^51^ A decrease in *RAD21* and *STAG1* contributes to senescence and loss of multipotency in MSCs.^52, 53^ Another important mediator, *EXO1* is involved in cell cycle and MMR, which have a role in telomere maintenance and proliferative capacity of stem cells.^54^ Our RT-PCR results verified the lower expression of *CCNA2*, *MCM6*, *RAD21*, *EXO1* and *STAG1* in P3 BMSCs compared with BMMNCs, indicating that expansion may alter telomerase activity and chromosomal stability of MSCs, leading to impaired stemness and multipotency. Many studies have demonstrated absence of telomerase activity in human MSCs.^55^ However, CCNA2, MCM6, RAD21, EXO1 and STAG1 mRNA expression was also reduced with expansion in human MSCs (Figure 7c). This also indicated that expansion of human MSCs affects multipotency by impairing cell cycle and chromosomal stabilization.

In conclusion, this study contradicted reports that early passage BMSCs are eligible for clinical application and demonstrated that unpurified BMMNCs have superior chondrogenic potential. In addition the expansion-dependent modulatory effects were highly associated with signaling pathways related to telomerase activity and chromosomal stability. This study indicates that *in vitro* culture is not necessary for stem cell-based therapy and non-artificial stem cells are the best choice.

## Materials and Methods

### Collagen preparation

Calf skin-derived type I collagen was prepared as previously described.^56, 57, 58, 59^ Briefly, collagen was purified via sodium chloride fractionation and fibril assembly after extraction from calf skin with pepsin and dissolved in 0.1% acetic acid. The collagen type I solution was stored at 4 °C. A 15 mg/ml solution was neutralized with 1 M NaOH.

### Surgical procedure

General anesthesia was performed via strictly aseptic techniques throughout the surgical procedures. To minimize the number of rabbits, both knees of the rabbits were subjected to the operation. The knee joint was opened laterally by dislocating the patellar tendon, and a cartilage-only defect (4-mm in diameter) was created on the patellar groove using a sharp biopsy punch. Then, a collagen hydrogel with encapsulated cells was injected into the defect. The cartilage defects were treated as follows: (i) filled with collagen only (*n*=60); (ii) press-fit filled with autologous P3 BMSCs embedded in collagen hydrogel (*n*=60); (iii) press-fit filled with freshly isolated autologous BMMNCs embedded in collagen hydrogel (*n*=60); and (iv) non-treated (*n*=12). A concentration of 10^7^ cells/ml was used in the cell-based therapy. The knee was closed with coated VICRYL 2.0 (Johnson & Johnson, Somerville, NJ, USA). Both analgesics and antibiotics were administered postoperatively. The animals were sacrificed by intravenous injection of a solution 4, 12, 26 and 52 weeks after surgery, and the repaired cartilage was harvested for evaluation.

### Gross observation and grading

The gross morphological evaluation of the engineered cartilage after 4, 12, 26 and 52 weeks of therapy was based on the scoring system proposed by Niederauer.^60^ The system comprises four major categories (‘edge integration,’ ‘smoothness of the cartilage surface,’ ‘cartilage surface, degree of filling’ and ‘color of cartilage, opacity or translucency of the neocartilage’) with a total possible score of 8.

### Magnetic resonance imaging

MRI of the repaired cartilage was performed at 26 weeks and 52 weeks after transplantation using a 2.0-Tesla Magnetom Espree MRI Scanner (Siemens, Erlangen, Germany) as described by Schüttler KF.^61^ All MR images were measured using the MOCART score^62^ by a senior musculoskeletal radiologist.

### Mechanical evaluation

Mechanical evaluation of engineered cartilage was performed as previously described.^63^ The compressive mechanical properties of the surface cartilage layer were assessed with a compression strength tester and software (model HY-0230; Shanghai Hengyi Instruments, China) using a 2-mm diameter cylindrical indenter fitted with a 10-N maximum loading cell. The unconfined equilibrium modulus was determined by applying a step displacement (20% strain) and monitoring the compressive force over time until equilibrium was reached. The crosshead speed used was ~0.06 mm/min. The ratio of equilibrium force to cross-sectional area was divided by the applied strain to calculate the equilibrium modulus (in MPa). Samples were tested following long-term treatment *in vivo* (26 and 52 weeks, *n*=6 samples per group) with native cartilage samples as the control (*n*=6).

### Western blot analysis

Total protein was extracted from engineered cartilage in RIPA Lysis Buffer (Beyotime, Beijing, China) and phenylmethanesulfonyl fluoride (PMSF) (Beyotime, China) following the manufacturer’s protocols. A total of 60 *μ*g of protein was loaded per lane and separated on a 6% polyacrylamide gel. The protein products were transferred to a PVDF membrane (Millipore, Billerica, MA, USA) by electroblotting. The membrane was blocked for nonspecific binding in 1% BSA blocking buffer (Boster, Wu Han, China), followed by incubation with primary antibodies targeting glyceraldehyde phosphate dehydrogenase (GAPDH), collagen type I (COL1A1) and collagen type II (COL2A1) (Boster) at 4 °C overnight. The membranes were washed and incubated with Alexa Fluor 790 dye-conjugated secondary antibodies (Invitrogen, Carlsbad, CA, USA) for 1 h. Finally, the membranes were visualized with an Odyssey Infrared Imaging System (LI-COR) according to the manufacturer’s instructions.

### Flow cytometry

CD44 (APC) and CD45 (APC) antibodies were purchased from AbDSerotec (Raleigh, NC, USA). CD90 (RPE) antibody was purchased from GenWayBiotec (San Diego, CA, USA). CD34 (FITC) and the isotype control antibodies were purchased from Becton-Dickinson (Franklin Lakes, NJ, USA). For surface marker studies, cells were washed with PBS and stained with a fluorochrome-conjugated primary antibody (CD34, CD44, CD45 and CD90) or an isotype control according to the manufacturer’s instructions. The samples were washed again and analyzed using a FACScan flow cytometer (Becton-Dickinson).

### Cell seeding in collagen hydrogel

Before seeding, freshly harvested BMMNCs and P3 BMSCs were counted using a Multisizer 3 Counter (Beckman Coulter, Boulevard Brea, CA, USA) to confirm approximately equivalent cell numbers. Briefly, cells were mixed with neutralized collagen solution at a concentration of 10^7^ cells/ml. Gelation occurred after 10 min at 37 °C. Cell-hydrogel composites were cultured in chondrogenic defined medium containing *α*-MEM (Gibco, Shanghai, China), 10% FBS, 1% Insulin-Transferrin-Selenium Solution (Gibco), 100 nM dexamethasone (Sigma Aldrich, Billerica, MA, USA), 50 *μ*g/ml ascorbic acid (Sigma) and 10 ng/ml TGF-*β*1 (Prope Tech, Rocky Hill, NJ, USA). The medium was replaced every 3 days. Samples were harvested at time points of 7, 14 and 21 days for various analyses and characterizations.

### Cell viability assay

Cell viability was measured at each time point using a LIVE/DEAD Viability/Cytotoxicity Kit (Thermo Fisher Scientific, Shanghai, China), with calcein-AM to identify live cells and PI to identify dead cells. The procedures followed the specifications of the kit. The stained cell-gel composites were observed via confocal laser scanning microscopy (Nikon A1, Tokyo, Japan).

### Staining the actin cytoskeleton

The cell-gel composites were washed with PBS and fixed with 4% paraformaldehyde at room temperature for 10 min. After washing with PBS, the samples were permeabilized with 0.5% Triton X-100 (Sigma Aldrich) and then stained for 30 min at room temperature with rhodamine-phalloidin (Invitrogen, USA) to visualize the actin cytoskeleton. Following three washes with PBS, the samples were mounted with Hoechst 33258 (Sigma Aldrich) and observed under a confocal laser scanning microscope (Nikon A1).

### Histological analysis

After culture *in vitro* for 7, 14 and 21 days, the cell-gel composites were embedded in paraffin and cut into 5-*μ*m sections. For the *in vivo* study, the engineered cartilage was decalcified using 10% ethylenediaminetetraacetic acid (EDTA) (Bostera) and subsequently embedded and cut into 5 *μ*m-thick sections perpendicular to the joint surface. The sections were stained with HE staining solution according to standard protocols. Safranin-O staining was performed to assess the synthesis and secretion of GAGs of composites *in vitro* and cartilage formation *in vivo*. Masson trichrome staining and immunohistochemical analysis for collagen type II (Boster) were also used to evaluate *in vivo* cartilage formation according to standard protocols. Images were captured under an upright microscope (Olympus BX53, Tokyo, Japan).

### Biochemical analysis

Cell-gel composites were washed with PBS and then treated with 60 *μ*g/ml proteinase K (Sigma) for 10 h at 60 °C. The digested aliquots were assayed separately to assess their DNA and GAG content. After staining with Hoechst 33258 (Sigma) for 5 min, the aliquots were analyzed in a microplate fluorescence reader (FLX800, Bio-tec, Burlington, Vermont, USA) at 460 nm; the absorbance value of the Hoechst 33258 dye was used as the baseline to determine the DNA content. The total GAG production was measured by the absorbance value after staining with 1,9-dimethylmethylene blue (DMMB) in a microplate reader (Thermo, Karlsruhe, Germany) at 525 nm. The calculated GAG production was normalized to the total DNA production to evaluate the biosynthetic activity of the cells in all groups.

### Telomerase activity

Telomerase activity was detected using a Telo TAGGG Telomerase PCR ELISA kit (Roche, Indianapolis, IN, USA) according to the manufacturer’s protocol (http://www.sigmaaldrich.com/catalog/product/roche/11854666910?lang=en&region=US). The activity of telomerase was measured with a microplate reader (Thermo) at 450 nm.

### Karyotype analysis

Cytogenetic evaluation using the G-banding method was conducted on BMMNCs and P3 BMSCs. For BMMNCs, 4 mg/kg of colchicine was injected into the abdominal cavity of rabbit before cell harvest. In addition, for P3 BMSCs, colchicine was added at a final concentration of 0.2 *μ*g/ml for 4 h at 37 °C. Cells were collected and resuspended in a hypotonic 0.075 mM KCl solution for 30 min at 37 °C. After centrifugation, cells were fixed with Carnoy’s solution (methanol: acetic acid=3:1). The cell suspension was air-dried and then stained with Giemsa stain solution (Solarbio, Beijing, China). Cells at metaphase were observed with Leica CytoVision (Pleasanton, CA, USA) platform.

### Microarray analysis and data processing

P3 BMSCs and BMMNCs were collected for microarray analysis before and after 21 days of 3D culture in collagen and chondrogenic supplements. Total RNA was isolated with TRIzol reagent (Invitrogen Life Technologies, USA). The RNA quantity was assessed using a NanoDrop ND-1000 spectrophotometer (NanoDrop, Hudson, NH, USA), and RNA integrity was assessed by standard denaturing agarose gel electrophoresis. Microarray analysis was performed by Shanghai KangChen Biotech on an Agilent Array platform in three replicates. The subsequent steps were performed according to the Agilent Whole Genome Oligo Microarray (one-color) protocol. Gene Ontology (GO) term analysis was performed using DAVID (http://david.abcc.ncifcrf.gov).^64^

Data preprocessing and normalization were performed using GeneSpring GX v12.1 (Shanghai, China). To select differentially expressed genes, ratio change threshold values of g2.0 or e0.05 were used. Hierarchical clustering was performed using log2-transformed data in Cluster 3.0, and heat maps were generated with the Treeview program. For comparative analysis, we used GeneSpring GX v12.1 to identify significantly differentially expressed genes between the P3 BMSCs and BMMNCs.

The differentially expressed genes were categorized by GO via the DAVID database. The P3 BMSC group was the control group in this study. Altered gene expression was compared with the expression of all genes on the array, and over-represented GO terms were identified automatically by the software with *P*<0.05 considered significant.^65^

We evaluated pathways with the greatest representation of gene expression that was significantly impacted by expansion based on KEGG pathway comments. Overrepresentation of genes in a KEGG pathway was present if a large portion of genes within that pathway were differentially expressed.^65^

### Real-time PCR analysis

Real-time PCR analysis was performed to detect chondrogenic differentiation-related genes in the cell-gel composites and engineered cartilage. Changes in the mRNA expression of aggrecan (*ACAN*), SRY-related high mobility group-box gene 9 (*SOX9*), *COL1A1* and *COL2A1*, and collagen type 10 (*COL10*) were investigated. To validate the microarray data, we examined the expansion-induced expression of several genes by comparing BMMNCs and P3 BMSCs. These genes include minichromosome maintenance complex component 6 (*MCM6*), stromal antigen 1 (*STAG1*), RAD21 cohesin complex component (*RAD21*), cyclin A2 (*CCNA2*) and exonuclease 1 (*EXO1*). Total RNA was extracted using TRIzol reagent (Invitrogen Life Technologies, USA) according to the manufacturer’s protocol. The subsequent steps were performed according to the manufacturer’s instructions. All primers (Supplementary Table S1) were designed based on established GenBank sequences, and the amplification of *GAPDH* was used as a control to assess PCR efficiency. The comparative Ct method was used for gene expression quantification.

### *In vitro* study of human BMMNCs and BMSCs

Human BMMNCs (hBMMNCs) and BMSCs (hBMSCs) were obtained from iliac crest marrow aspirates from patients participating in the clinical trial (collagen scaffold-based autologous BMMNC therapy of knee focal cartilage defects) carried out in the First Affiliated Hospital of Guangxi Medical University (Nanning, China). The chondrogenic potential of hBMMNCs and third passage hBMSCs (P3 hBMSCs) was evaluated with biochemical assays and RT-PCR as described above. In addition, the telomerase activity and associated mRNA (*CCNA2*, *MCM6*, *RAD21*, *EXO1* and *STAG1*) expression in hBMMNCs and P3 hBMSCs were also detected.

### Statistical analysis

Statistical analysis was performed using GraphPad Prism 5.0 (GraphPad Software, La Jolla, CA, USA). Data were evaluated by Student’s *t*-test, one-way ANOVA using SPSS 16.0 (IBM Armonk, NY, USA). Data are represented as means±S.D. and distributed approximately normally.

## Figures and Tables

**Table 1:** KEGG signaling pathways downregulated by MSCs expanded *in vitro*

**Pathway title**	**Count**	**Genes downregulated**
Cell cycle	33	*ANAPC1, ANAPC5, ATR, BUB1, CCNA2, CCNB2, CCND3, CCNE1, CCNE2, CDC14A, CDC23, CDC6, CDK2, CDK4, CDK6, MCM3, MCM5, MCM6, MYC, PRKDC, RAD21, RBL2, SMAD2, SMC3, STAG1, STAG2, TFDP1, TGFB1, TP53, TTK, WEE1, YWHAH, YWHAQ*
Mismatch repair	10	*EXO1, LIG1, MLH1, MSH3, MSH6, PMS2, POLD2, POLD3, RFC5, RPA1*
DNA replication	12	*FEN1, LIG1, MCM3, MCM5, MCM6, POLA1, POLA2, POLD2, POLD3, POLE, RFC5, RPA1*

Threshold: *P*-value<0.05.
