# Correction: In vitro expansion impaired the stemness of early passage mesenchymal stem cells for treatment of cartilage defects

**DOI:** 10.1038/s41419-019-1939-9

**Published:** 2019-09-26

**Authors:** Tongmeng Jiang, Guojie Xu, Qiuyan Wang, Lihui Yang, Li Zheng, Jinmin Zhao, Xingdong Zhang

**Affiliations:** 1 Guangxi Engineering Center in Biomedical Materials for Tissue and Organ Regeneration, The First Affiliated Hospital of Guangxi Medical University, Nanning, 530021 China; 2 Department of Orthopaedics Trauma and Hand Surgery, The First Affiliated Hospital of Guangxi Medical University, Nanning, 530021 China; 3 Center for Genomic and Personalized Medicine, Guangxi Medical University, Nanning, 530021 China; 4 School of Nursing, Guangxi Medical University, Nanning, 530021 China; 5 Collaborative Innovation Center of Guangxi Biological Medicine, The First Affiliated Hospital of Guangxi Medical University, Nanning, 530021 China; 6 Guangxi Key Laboratory of Regenerative Medicine, The First Affiliated Hospital of Guangxi Medical University, Nanning, 530021 China; 7 National Engineering Research Center for Biomaterials, Sichuan University, Chengdu, 610064 China

**Correction to:** Cell Death and Disease

10.1038/cddis.2017.215 published online 01 June 2017

Following the publication of this article, the authors noticed that Fig. 5c in the original paper is incorrect due to an error in image handling during the creation of the figure. Specifically, the image for the group P3 BMSCs of time point 21d and the image in the group BMMNCs of time point 14d were incorrect. These linked to the group for BMMNCs of time point 21d, which was also, as a result, incorrect. The correct figure is shown below. The error does not impact the conclusions of the article. The authors would like to apologize for any inconvenience this may have caused.

**Fig. 5:**
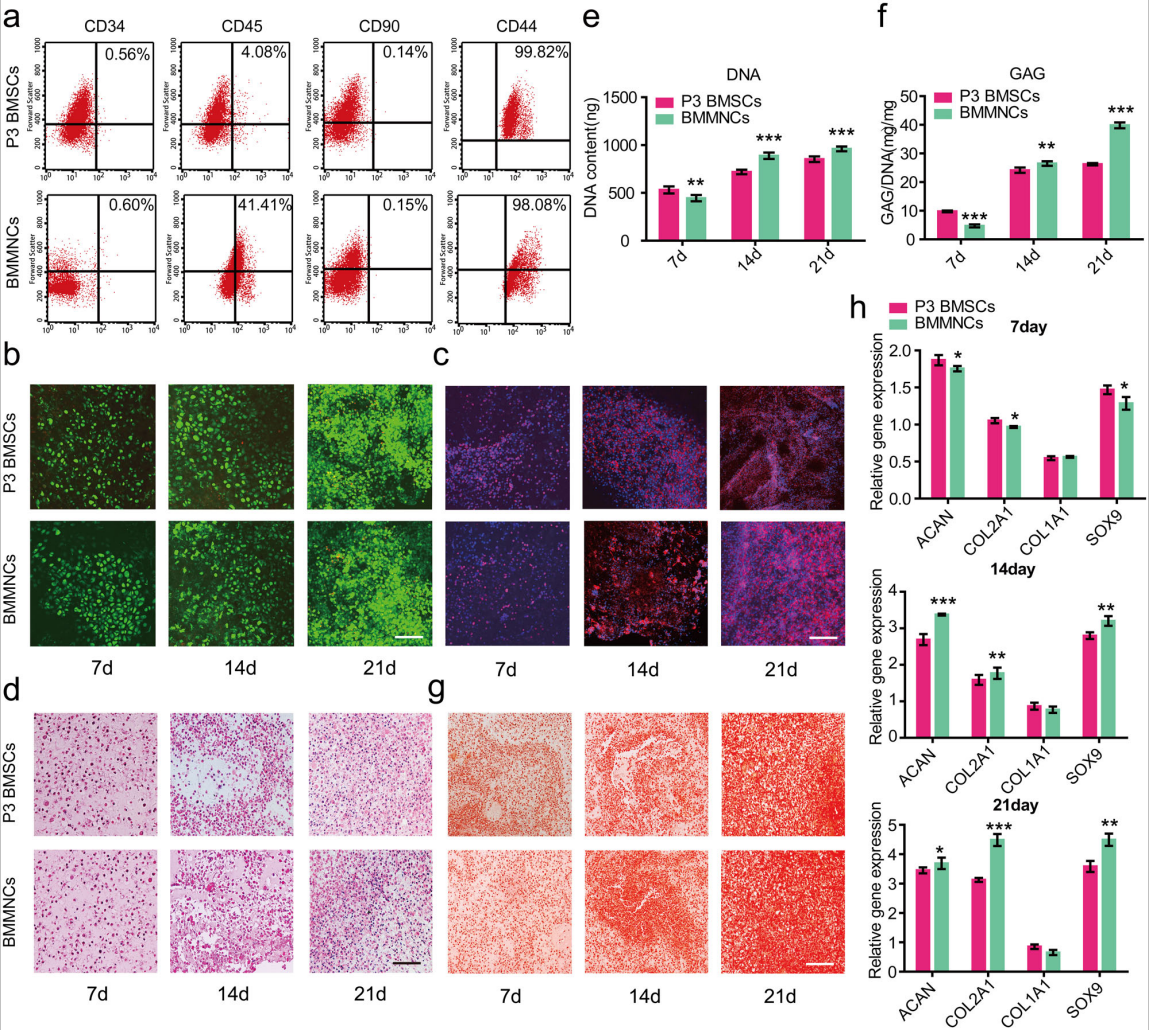
▓

