# Supplementary material for: In vitro expansion impaired the stemness of early passage mesenchymal stem cells for treatment of cartilage defects
Source: Cell Death Dis. 2017 Jun 1;8(6):e2851–. doi: 10.1038/cddis.2017.215 (PMC5520885; doi:10.1038/cddis.2017.215)
Supplement: Supplementary Information [file cddis2017215x1.pdf]

Supplemental Table 1. Sequences of primers used in real-time PCR

Species	Genes	Primer sequences (5′ →3′)	Size （bp）
Rabbit	GAPDH	GTCATCATCTCAGCCCCCTC<br>GGATGCGTTGCTGACAATCT	99
	COL1A1	GCGGTGGTTACGACTTTGGTT<br>AGTGAGGAGGGTCTCAATCTG	139
	COL2A1	CAGGCAGAGGCAGGAAACTAAC<br>CAGAGGTGTTTGACACGGAGTAG	132
	COL10	ATCAGCCACTGGGAAGCC<br>TTCGGTCCACTTGGTCCTC	72
	ACAN	ATGGCTTCCACCAGTGCG<br>CGGATGCCGTAGGTTCTCA	127
	SOX9	GTACCCGCACCTGCACAAC<br>TCCGCCTCCTCCACGAAG	100
	CCNA2	TGACTTAGCTGCACCAACGG<br>CCCGTGACTGTGTAGAGTGC	195
	MCM6	CAAGACAGGGACATCGCACT<br>GAGGTCCAGGCATGGTAAGG	178
	RAD21	GACTCACCCAGTTCACCCAG<br>CACAAACTGGGTTTCGGCAG	128
	STAG1	TTGCGAACATCGGGACATCT<br>GCTGAATTGTTGGGCCACAT	246
	EXO1	CAGCTTCTTCGTGAGGGGAA<br>TCCCTGAGACCTAGCAGCTT	111
human	GAPDH	CTATAAATTGAGCCCGCAGC<br>GACCAAATCCGTTGACTCCG	143
	COL1A1	GCTTCACCTACAGCGTCACT<br>AAGCCGAATTCCTGGTCTGG	154
	COL2A1	TGAGCCATGATTCGCCTCG<br>CCCTTTGGTCCTGGTTGCC	110
	COL10	CGCTGAACGATACCAAATGCC<br>TTCCCTACAGCTGATGGTCC	258
	ACAN	CTACACGCTACACCCTCGAC<br>ACGTCCTCACACCAGGAAAC	216
	SOX9	AAGCTCTGGAGACTTCTGAACG<br>CGTTCTTCACCGACTTCCTCC	133
	CCNA2	CTGGTGGTCTGTGTTCTGTGA<br>TGCCAGTCTTACTCATAGCTGA	137
	MCM6	CCAGTGGTAAAGCGTCCAGT<br>CCACTGATTGGGTTTGCTGC	268
	RAD21	GCTGCCGCAAAGTTCTACAG<br>GGTCCAGGTGTTGCGATGAT	101
	STAG1	ACAGATGCTGTACACCGTTCA<br>GGCAGGGCATGAAATAAGTGG	183

EXO1	AAACCTGAATGTGGCCGTGT<br>CCTCATTCCCAAACAGGGACT	113

Supplementary figures

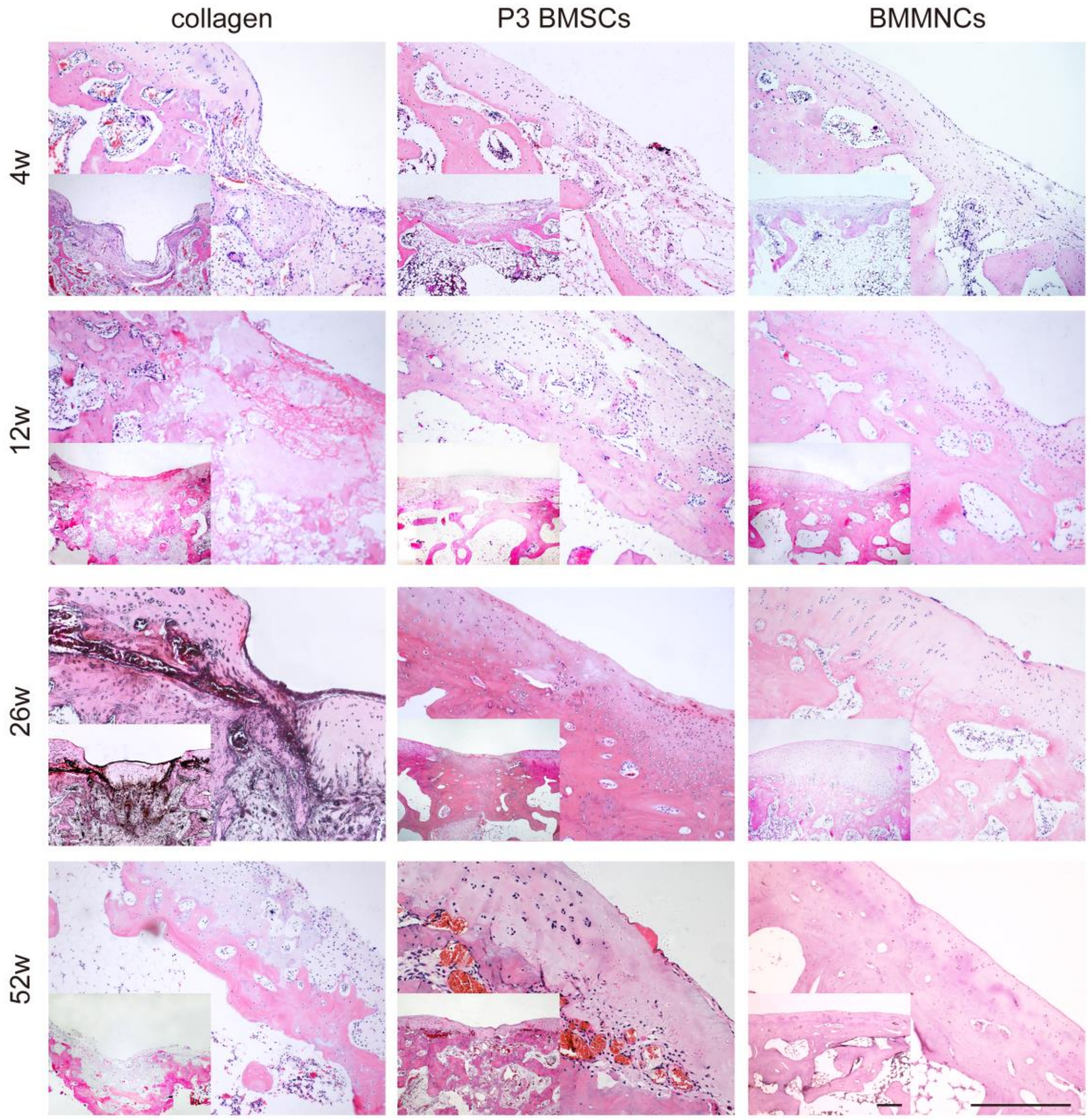

Figure. S1 HE staining of repaired cartilage *in vivo* at different post-transplantation time, respectively. Scale bar: 400μm.

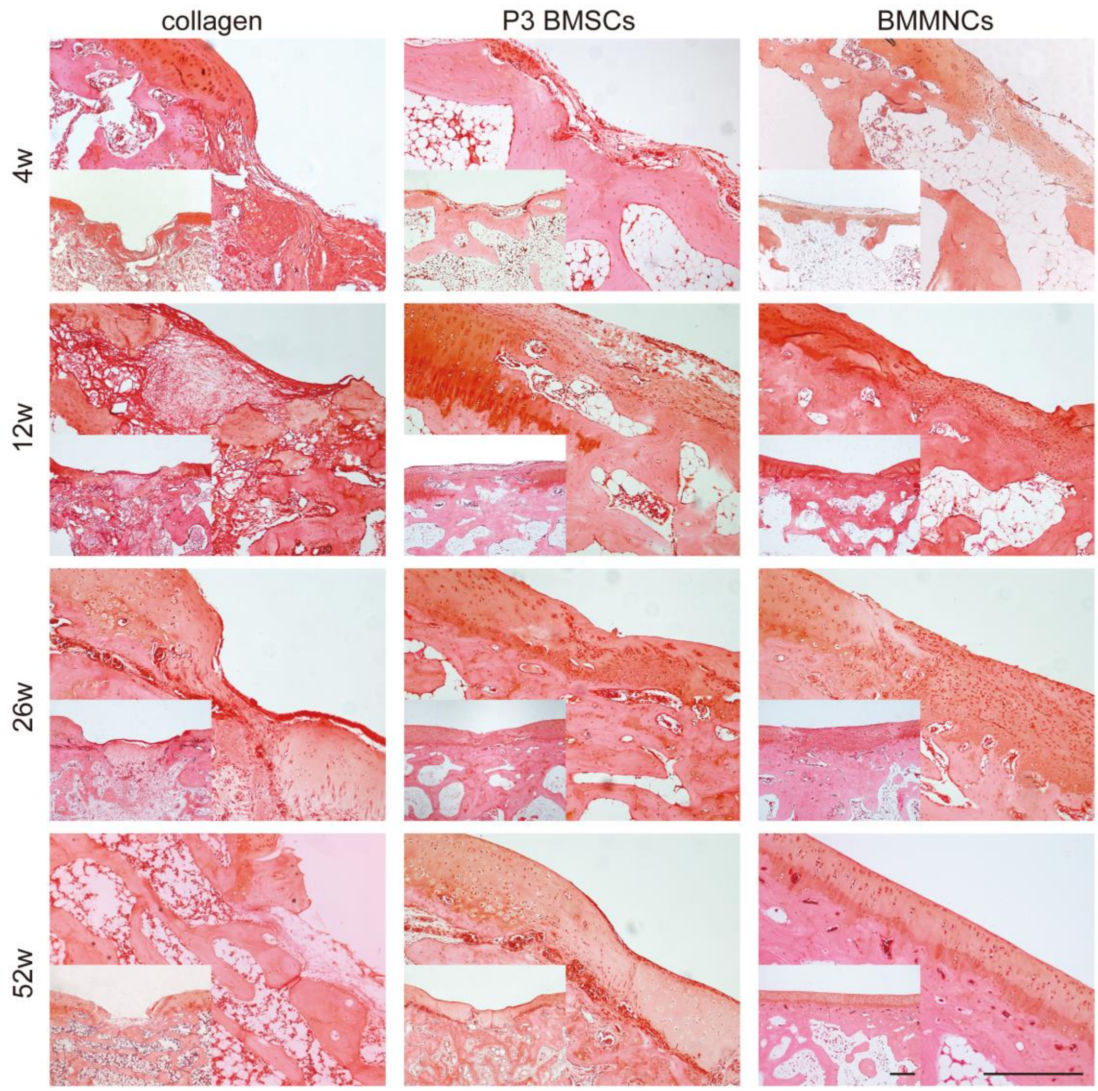

Figure. S2 Safranin-O staining of repaired cartilage *in vivo* at different post-transplantation time, respectively. Scale bar: 400µm.
